# Durability of benralizumab effectiveness in severe eosinophilic asthma patients with and without chronic rhinosinusitis with nasal polyps: a *post hoc* analysis from the ANANKE study

**DOI:** 10.3389/falgy.2025.1501196

**Published:** 2025-03-20

**Authors:** Luisa Brussino, Maria Aliani, Elena Altieri, Pietro Bracciale, Maria Filomena Caiaffa, Paolo Cameli, Giorgio Walter Canonica, Cristiano Caruso, Stefano Centanni, Fausto De Michele, Stefano Del Giacco, Fabiano Di Marco, Laura Malerba, Francesco Menzella, Girolamo Pelaia, Paola Rogliani, Micaela Romagnoli, Pietro Schino, Jan Walter Schroeder, Gianenrico Senna, Alessandra Vultaggio, Maria D’Amato

**Affiliations:** ^1^ Dipartimento di Scienze Mediche, Università degli Studi di Torino; SCDU Immunologia e Allergologia, AO Ordine Mauriziano Umberto I, Torino, Italy; ^2^ UO Pneumologia e Pneumologia Riabilitativa, ICS Maugeri, IRCCS Bari, Bari, Italy; ^3^ Reparto di Pneumologia, P.O. Garbagnate Milanese, Garbagnate Milanese (MI), Italy; ^4^ Reparto di Pneumologia, Ospedale Ostuni, Ostuni (BR), Italy; ^5^ Cattedra e Scuola di Allergologia e Immunologia Clinica, Dipartimento di Scienze Mediche, Università di Foggia, Foggia, Italy; ^6^ Respiratory Diseases Unit, Department of Medicine, Surgery and Neurosciences, University of Siena, Siena, Italy; ^7^ Department of Biomedical Sciences, Humanitas University, Pieve Emanuele (MI), Italy; ^8^ Personalized Medicine Center: Asthma and Allergology, Humanitas Research Hospital, Rozzano (MI), Italy; ^9^ UOSD Allergy and Clinical Immunology, Fondazione Policlinico A. Gemelli, IRCCS, Rome, Italy; ^10^ UOC Internal Medicine, Ospedale Isola Tiberina - Gemelli Isola, Rome, Italy; ^11^ Respiratory Unit, ASST Santi Paolo e Carlo, Department of Health Sciences, Università degli Studi di Milano, Milano, Italy; ^12^ UOC Pneumologia e Fisiopatologia Respiratoria, AORN A. Cardarelli, Napoli, Italy; ^13^ Dipartimento di Scienze Mediche e Sanità Pubblica, Università degli Studi di Cagliari, Monserrato, Cagliari, Italy; ^14^ Department of Health Sciences, Università degli Studi di Milano, Pneumologia, ASST Papa Giovanni XXIII, Bergamo, Italy; ^15^ Medical Affairs R&I, AstraZeneca, Milano, Italy; ^16^ Pulmonology Unit, Ospedale “S. Valentino”, AULSS 2 Marca Trevigiana, Montebelluna (TV), Italy; ^17^ Dipartimento di Scienze della Salute, Università Magna Graecia, Catanzaro, Italy; ^18^ Division of Respiratory Medicine, University Hospital “Fondazione Policlinico Tor Vergata”, Rome, Italy; ^19^ Unit of Respiratory Medicine, Department of Experimental Medicine, University of Rome “Tor Vergata”, Roma, Italy; ^20^ UOC Pneumologia, AULSS 2 Marca Trevigiana, Treviso, Italy; ^21^ Fisiopatologia Respiratoria, Ospedale Generale Regionale, Ente Ecclesiastico “F. Miulli”, Acquaviva delle Fonti (BA), Italy; ^22^ Allergy and Clinical Immunology, ASST Grande Ospedale Metropolitano Niguarda, Milano, Italy; ^23^ Allergy Unit and Asthma Center, Verona University Hospital, Verona, Italy; ^24^ Dipartimento di Medicina Sperimentale e Clinica, Università degli Studi di Firenze, Firenze, Italy; ^25^ UOSD Malattie Respiratorie “Federico II”, Ospedale Monaldi, AO Dei Colli, Napoli, Italy

**Keywords:** benralizumab, asthma, eosinophils, CRSwNP, long-term

## Abstract

**Introduction:**

Severe eosinophilic asthma (SEA) often co-occurs with chronic rhinosinusitis with nasal polyps (CRSwNP), worsening asthma symptoms. Earlier studies have shown that benralizumab improves asthma outcomes with greater efficacy if patients present CRSwNP.

**Methods:**

This *post hoc* analysis of the ANANKE study (NCT04272463) reports data on the long-term effectiveness of benralizumab between SEA patients with and without CRSwNP (*N* = 86 and *N* = 75, respectively) treated for up to 96 weeks.

**Results:**

Before benralizumab initiation, CRSwNP patients displayed longer SEA duration, greater oral corticosteroid (OCS) use and blood eosinophil count. After 96 weeks of treatment, the annual exacerbation rate (AER) decreased in both groups, with CRSwNP patients achieving considerable reductions than No-CRSwNP patients (severe AER dropped by 100% and 95.6%, respectively). While lung function improvement was comparable at week 96, CRSwNP patients showed a faster response to benralizumab, with a rise of forced expiratory volume in 1 s (FEV_1_) at 16 weeks that was maintained throughout the study. Median OCS daily dose decreased to 0.0 mg in both groups at 96 weeks, but benralizumab OCS-sparing effect was faster in CRSwNP patients (median OCS dose was 0.0 mg and 2.5 mg in CRSwNP and No-CRSwNP patients respectively, at 48 weeks). Although asthma control test (ACT) median scores were comparable, greater proportions of CRSwNP patients displayed well-controlled asthma (ACT ≥ 20) than No-CRSwNP patients at all time points.

**Discussion:**

These findings show benralizumab long-term effectiveness in SEA patients with and without CRSwNP, highlighting its superior and faster-acting benefits on asthma outcomes in presence of CRSwNP.

## 1 Introduction

Severe asthma (SA) is a heterogeneous chronic condition of the airways that can be accompanied by multiple comorbidities, among which chronic rhinosinusitis with nasal polyps (CRSwNP) is particularly frequent. The clinical manifestations of CRSwNP include nasal congestion, loss of smell, rhinorrhea, facial pain, headache, and sleep difficulties, which further impair asthma patients' quality of life (QoL) (1–3). The coexistence of SA and CRSwNP in the same patient implies common pathophysiological mechanisms spreading across both lower and upper airways; accordingly, asthma patients are more likely to develop CRSwNP and vice versa (4, 5). The theory of coexisting asthma and CRSwNP being a single disease, referred to as “united airways disease” (UAD), is widely embraced within the scientific community and warrants the need for a multidisciplinary approach to effectively manage comorbid patients (6, 7).

Severe eosinophilic asthma (SEA) is one of the most challenging SA pheno-endotype to treat. It is characterized by a pronounced type 2 (T2) inflammatory response driven by the activation of T helper (Th)2 cells and type 2 innate lymphoid cells (ILC2). These cells secrete elevated levels of T2 cytokines (IL4, IL5, IL13), which create a positive feedback loop by continuously recruiting eosinophils and perpetuating the inflammatory cascade in the airways (8, 9). As a result, SEA is characterized by severe symptoms, such as threatening exacerbations and progressive airflow decline (10–12). In the presence of CRSwNP, reported in 40%–60% of SEA patients, asthma symptoms are usually exacerbated and become more difficult to control, leading to a substantial worsening in the quality of life (QoL) (13–15).

Benralizumab is the only monoclonal antibody (mAb) currently approved for SEA treatment that targets the IL5 receptor alpha (IL5Rα) and induces eosinophils apoptosis via a unique mechanism of afucosylation-enhanced Ab-dependent cell-mediated cytotoxicity (ADCC) (16, 17). Apart from its anti-eosinophilic activity, benralizumab has been found to mediate additional immune-modulatory processes, such as modifications in the number of CD3+ T cell subsets and activation of NK cells, that contribute to ameliorate asthma symptoms (18). Importantly, benralizumab has been associated with superior effects in reducing asthma exacerbations, enhancing respiratory function and improving QoL in SEA and CRSwNP comorbid patients, as demonstrated by results from the randomized clinical trial (RCT) ANDHI (19) and *post hoc* analyses from the pivotal studies SIROCCO and CALIMA (20). Real-life studies have confirmed the enhanced effectiveness of benralizumab in SEA patients with CRSwNP compared to those without CRSwNP (21–23). Recently, Pelaia et al. reported a higher prevalence of CRSwNP in SEA patients achieving sustained clinical remission after two years of benralizumab treatment. Notably, CRSwNP emerged as a significant predictor of remission probability. Importantly, benralizumab also demonstrated significant improvements in CRSwNP outcomes. These results further support the role of CRSwNP in mediating the favourable, long-term response of SEA patients to benralizumab (24).

Consistently with the growing body of evidence, the international guidelines Global Strategy for Asthma Management (GINA) recognize CRSwNP as a predictor of benralizumab enhanced efficacy in controlling asthma (25).

ANANKE (NCT04272463) is a retrospective, multicenter Italian study in which the characteristics and clinical outcomes of SEA patients treated with benralizumab have been described (26). The differences between SEA patients with and without CRSwNP were also evaluated, revealing that comorbid patients were characterized by a higher blood eosinophil count (BEC) and a greater OCS use compared with patients without CRSwNP. After a median treatment period of 9.8 months, benralizumab treatment improved all clinical outcomes in both groups, with a more pronounced effect in reducing any and severe AER and decreasing OCS use observed in CRSwNP patients (27). Long-term data on the effectiveness of benralizumab (up to 96 weeks) have also been published, highlighting the durable response to the biologic (28). In this novel *post hoc* analysis, we report data on the effectiveness of benralizumab in SEA patients with and without CRSwNP, treated for up to 96 weeks.

## 2 Material and methods

### 2.1 Study design

ANANKE (NCT04272463) is an Italian multicenter, observational, retrospective study whose methods have been extensively described (26). As explained by Vultaggio et al. (28), the observation period was extended up to 96 weeks, and patients signed the additional and amended study informed consent and privacy forms. During the observation period, data were collected at 4, 16, 24, 48, and 96 weeks after benralizumab initiation (i.e., the index date). ANANKE was performed following the principles of the Declaration of Helsinki, as well as the laws and guidelines regulating Italian medical practice. The ethics committees/institutional review boards of the participating centers approved the study.

### 2.2 Study population

Inclusion and exclusion criteria have been previously detailed (26). Patients were adults (≥18 years old) affected by SEA and requiring treatment with high doses of inhaled corticosteroids (ICS) and a long-acting beta2-agonist (LABA), with or without additional asthma controllers. Benralizumab treatment was initiated at least 3 months before patients' enrollment. Patients were excluded if they participated in other studies with a specific patient management strategy that differed from the site's normal clinical practice.

Eligible patients satisfied all inclusion, exclusion, and amendment criteria. Evaluable patients comprehended all eligible patients with BEC data available at the index date.

### 2.3 Outcomes and variables

Data were recorded in the electronic case report form (eCRF) after being collected from hospital medical charts.

The primary endpoint aimed to characterize the socio-demographic and clinical characteristics of the two groups of patients at the index date. As previously specified (26), data at the index date were gathered during the 12 months before benralizumab initiation. Among patients' exacerbations, severe exacerbations were distinguished from other exacerbations based on the need for at least one of the following: (a) treatment with systemic corticosteroids for 3 days or more, or an increase in the dosage of maintenance OCS; (b) an emergency department or urgent care visit during which systemic corticosteroids were administered; (c) hospitalization.

Secondarily, the variation in the following clinical and laboratory outcomes was assessed during benralizumab treatment: BEC, AER (any and severe), proportion of patients without any exacerbations, whether mild or severe, predicted forced expiratory volume in the first second (FEV_1_), measured pre-bronchodilator (BD), OCS use and daily dosage (expressed as prednisone-equivalent), proportion of patients reducing or permanently interrupting OCS, ACT score, the proportion of patients reaching ACT score ≥20 (well-controlled asthma).

Measurements were taken at the index date and after 48 and 96 weeks of treatment; data at earlier time points (4, 16, 24, weeks) were included when available.

### 2.4 Statistical analysis

In line with previous ANANKE studies, statistical analyses were descriptive only and were performed among patients with CRSwNP and without CRSwNP. Data are expressed as either mean ± standard deviation (SD), median [interquartile range (IQR)], or absolute numbers and frequencies. Demographic and clinical characteristics at the index date were assessed in evaluable patients; secondary endpoints were assessed in evaluable patients for secondary analyses at 48 and 96 weeks.

## 3 Results

### 3.1 Patients disposition and characteristics at index date

Patient disposition in the ANANKE study has been previously detailed (28). Briefly, 218 SEA patients were enrolled in the ANANKE study between December 2019 and July 2020, and 167 patients were eligible for analysis at 96 weeks. For this *post hoc* analysis, SEA patients were divided into two groups, CRSwNP and No-CRSwNP. Among these, evaluable patients with consistent data were *N* = 161 (CRSwNP, *N* = 86; No-CRSwNP, *N* = 75) at the index date, *N* = 153 (CRSwNP, *N* = 79; No-CRSwNP, *N* = 74) at 48 weeks, and *N* = 113 (CRSwNP, *N* = 59; No-CRSwNP, *N* = 54) at 96 weeks.

Table 1 shows socio-demographic and clinical characteristics of CRSwNP and No-CRSwNP patients at the index date. Female patients were more prevalent in the No-CRSwNP group than in the CRSwNP group (74.7% and 50%, respectively). The two groups were comparable in terms of mean age (56 and 58 years old in CRSwNP and No-CRSwNP, respectively) and mean age at asthma onset (38 and 41 years in CRSwNP and No-CRSwNP, respectively). CRSwNP patients experienced a longer median duration of asthma compared with No-CRSwNP patients (15.8 and 11.9 years in CRSwNP and No-CRSwNP, respectively); consistently, they were also characterized by a longer median duration of SEA (2.1 and 1.6 years in CRSwNP and No-CRSwNP, respectively). Analysis of relevant comorbidities besides CRSwNP revealed similar prevalences between CRSwNP (69.8%) and No-CRSwNP (72%) groups, with eosinophilic granulomatosis with polyangiitis (EGPA) abeing more frequent among CRSwNP patients than No-CRSwNP patients (9.3% and 0.0%, respectively). A higher percentage of patients with BMI above 30 was found in the No-CRSwNP group (22.7%) compared to the CRSwNP group (9.3%). Median BEC was slightly higher in the CRSwNP group [600 (460–800) cells/mm^3^] when compared to the No-CRSwNP patients [525 (355–960) cells/mm^3^] whereas median IgE levels were similar (CRSwNP, 198.5 IU/ml; No-CRSwNP, 227.5 IU/ml).

**Table 1:** Socio-demographic and clinical characteristics of CRSwNP and No-CRSwNP patients at the index date. Data were collected at the index date (either at benralizumab initiation or during the previous 12 months) and are expressed as *N* (%), mean ± SD, or median (IQR). Unless otherwise stated, evaluated patients were *N* = 86 and *N* = 75 in the CRSwNP and No-CRSwNP groups, respectively.

Characteristics at index date	CRSwNP (*N* = 86)	No-CRSwNP (*N* = 75)
Age (years)	56 ± 12.9	58 ± 12.3
Females	43 (50.0%)	56 (74.7%)
Males	43 (50.0%)	19 (25.3%)
BMI categories		
Underweight	1 (1.2%)	1 (1.3%)
Normal	33 (38.4%)	23 (30.7%)
Overweight	33 (38.4%)	29 (38.7%)
Obese	8 (9.3%)	17 (22.7%)
Unknown	11 (12.8%)	5 (6.7%)
Smoking status		
Non-smoker	57 (66.3%)	49 (65.3%)
Current smoker	2 (2.3%)	3 (4.0%)
Previous smoker	24 (27.9%)	19 (25.3%)
Unknown	3 (3.5%)	4 (5.3%)
Smoking duration (years) (*N* = 17, *N* = 13)	14.0 ± 9.0	15.0 ± 10.5
Asthma duration (years) (*N* = 86, *N* = 74)	15.8 (9.5–25.4)	11.9 (7.8–26.4)
Age at diagnosis (years) (*N* = 86, *N* = 74)	38.0 (24.0–49.0)	41.0 (30.0–51.0)
SEA duration (years) (*N* = 85, *N* = 72)	2.1 (1.0–4.5)	1.6 (1.0–4.1)
Age at diagnosis (years) (*N* = 85, *N* = 72)	52.0 (43.0–62.0)	55.0 (46.0–63.0)
Atopy		
No	47 (54.7%)	38 (50.7%)
Yes	39 (45.3%)	37 (49.3%)
BEC (cells/mm^3^)	600 (470–800)	525 (355–960)
Total serum IgE (IU/ml) (*N* = 46, *N* = 44)	198.5 (117–474)	227.5 (59.5–532.5)
Any comorbidities (excluding CRSwNP)	60 (69.8%)	54 (72.0%)
≥1 any asthma-related	51 (59.3%)	39 (52.0%)
≥1 current OCS-related	33 (38.4%)	31 (41.3%)
≥1 other ongoing	11 (14.7%)	15 (17.4%)
OCS for asthma treatment	25 (29.1%)	16 (21.3%)
OCS daily dose (mg) (*N* = 23, *N* = 16)	10.0 (5.0–25.0)	6.0 (5.0–10.0)
Exacerbations (*N* = 82, *N* = 71)		
Patients with ≥1, any	74 (90.2%)	69 (97.2%)
Patients with ≥1, severe	26 (31.7%)	30 (42.3%)
Any AER	3.78	4.52
Severe AER	0.84	1.14
Lung function		
Pre-BD FEV_1_ (L) (*N* = 79, *N* = 64)	2.1 (1.6–2.6)	1.7 (1.7–2.3)
Pre-BD FEV_1_ predicted (%) (*N* = 79, *N* = 64)	72 (55–89)	67 (47–81)
Pre-BD FVC (L) (*N* = 79,64)	3.2 (2.5–3.9)	2.6 (2.0–3.2)
Pre-BD FEV_1_/FVC at index date (*N* = 55, *N* = 51)	0.7 (0.6–0.7)	0.7 (0.6–0.8)
FeNO (ppb) (*N* = 28, *N* = 22)	62.5 (37.5–81.5)	22 (18–45)
ACT score (*N* = 79, *N* = 64)	15 (12–19)	14 (11.5–16.5)

Lung function appeared to be slightly higher in CRSwNP patients, with a median pre-BD FEV_1_ volume of 2.1 L (vs. 1.7 L in No-CRSwNP patients) and median pre-BD FEV_1_ predicted of 72.0% (vs. 67.0% in No-CRSwNP patients). FeNO levels were higher in CRSwNP patients than in No-CRSwNP patients (62.5 ppb vs. 22.0 ppb).

No dissimilarity was observed in terms of asthma control between the two groups, measured via the ACT score [CRSwNP: 15.0 (12.0–19.0); No-CRSwNP, 14.0 (11.5–16.5)]. Similarly, there were only modest disparities in exacerbation frequency between CRSwNP and No-CRSwNP groups (any AER, 3.78 and 4.52 respectively; severe AER, 0.84 and 1.14, respectively). However, more patients in the No CRSwNP group experienced at least one or more exacerbations of any severity in the previous year (*N* = 74, 90.2%) compared to patients in the CRSwNP group (*N* = 69, 97.2%).

A higher percentage of CRSwNP patients used OCS to control their asthma compared with No-CRSwNP patients (29.1% and 21.3%, respectively); CRSwNP patients also used a higher median OCS dosage than No-CRSwNP patients (10.0 mg and 6.0 mg, respectively).

### 3.2 Long-term reduction of exacerbations

**Figure 1:**
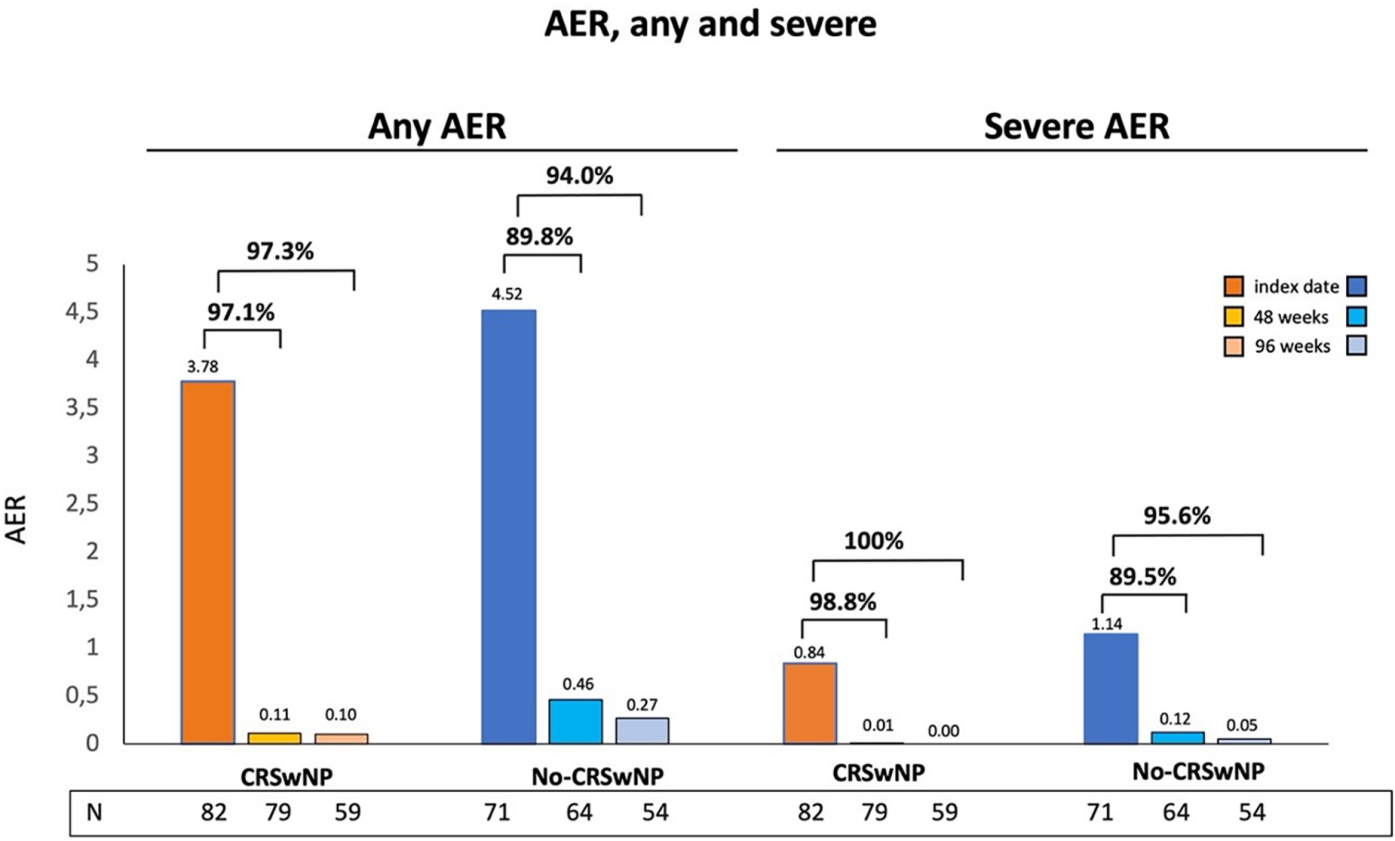
AER reduction during benralizumab treatment in CRSwNP and No-CRSwNP patients. Any and severe AER are shown at index date and after 48 and 96 weeks of treatment with benralizumab.

During this period, benralizumab demonstrated a substantial reduction in exacerbations in both populations, with a more pronounced decrease observed in the CRSwNP population, as early as the 48th week (Figure 1). Among CRSwNP patients, any AER drastically decreased from 3.78 to 0.11 at 48 weeks, resulting in a 97.1% reduction. Any AER remained low over the entire observation period, reaching the value of 0.10 at 96 weeks (97.3% reduction). In No-CRSwNP patients, the AER, higher at baseline compared to the CRSwNP group, decreased significantly at 48 weeks, but less markedly, with an overall reduction of 89.8% (from 4.52 to 0.46). By 96 weeks, any AER further decreased to 0.27, reflecting a 94.0% reduction.

CRSwNP patients also showed a prominent decline in severe AER, which decreased from 0.84 to 0.01 at 48 weeks (98.8% reduction), and dropped to 0.0 at 96 weeks (100% reduction). In No-CRSwNP patients, severe AER decreased from 1.14 to 0.12 at 48 weeks (89.5% reduction) and reached 0.05 at 96 weeks (95.6% reduction) (Figure 1).

**Figure 2:**
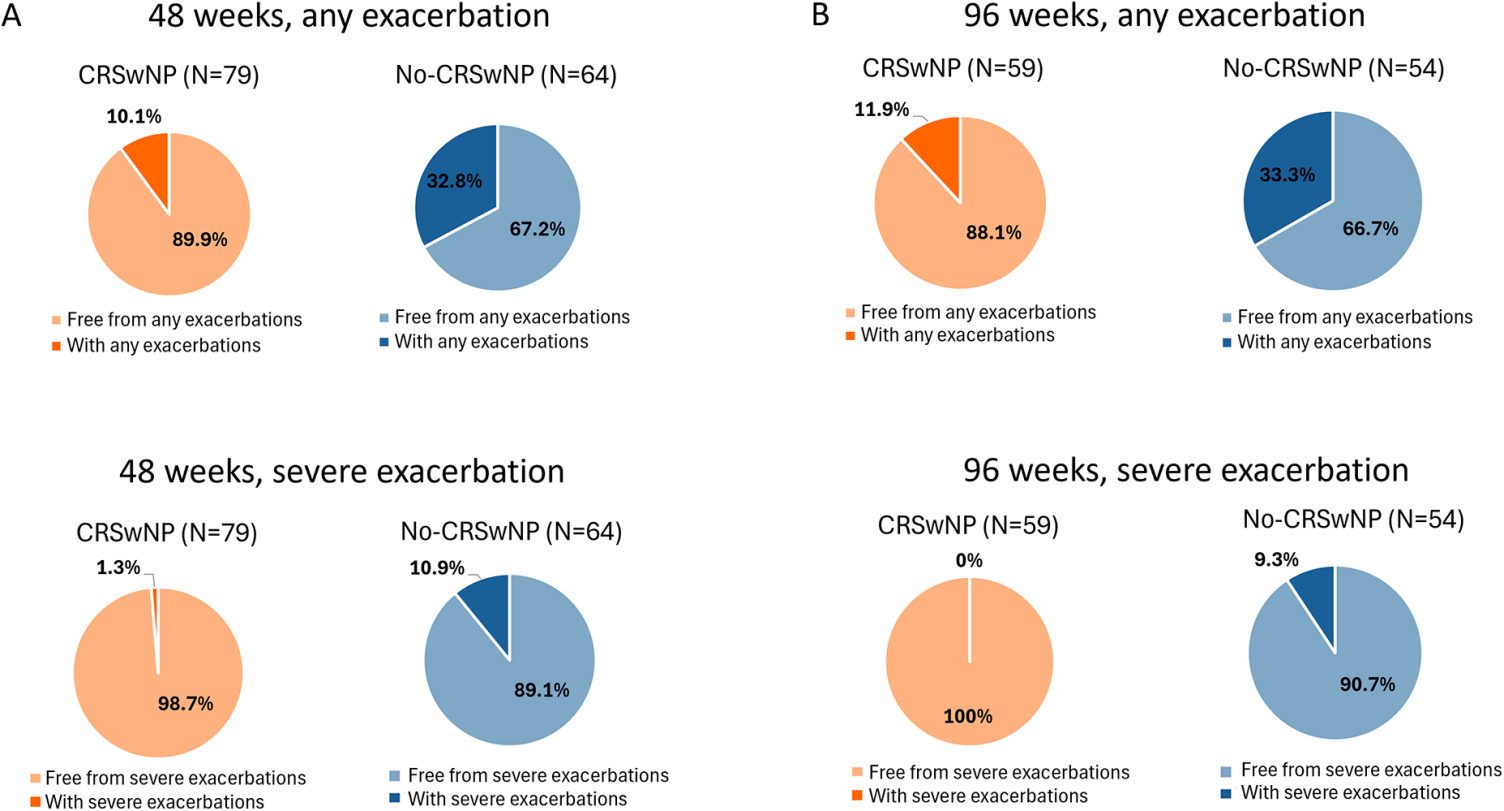
Percentages of CRSwNP and No-CRSwNP patients with and without any or severe exacerbations during benralizumab treatment. Data are shown after 48 **(A)** and 96 weeks **(B)** of treatment with benralizumab.

The proportion of patients free from any and severe exacerbations, as well as patients who still experienced exacerbations during benralizumab treatment, were also assessed. As shown in Figure 2A, at 48 weeks, 89.9% of CRSwNP patients and 67.2% of No-CRSwNP patients experienced no exacerbations, with 98.7% and 89.1% of them being free from severe exacerbations, respectively. By week 96, 88.1% of CRSwNP patients were free from any exacerbation, and 100% were free from severe exacerbations. In contrast, 66.7% of No-CRSwNP patients were free from any exacerbations, and 90.7% were free from severe exacerbations (Figure 2B).

A nearly complete depletion of BEC was observed in both CRSwNP and No-CRSwNP groups at 96 weeks post-treatment initiation with benralizumab (data not shown).

### 3.3 Lung function improvement

**Figure 3:**
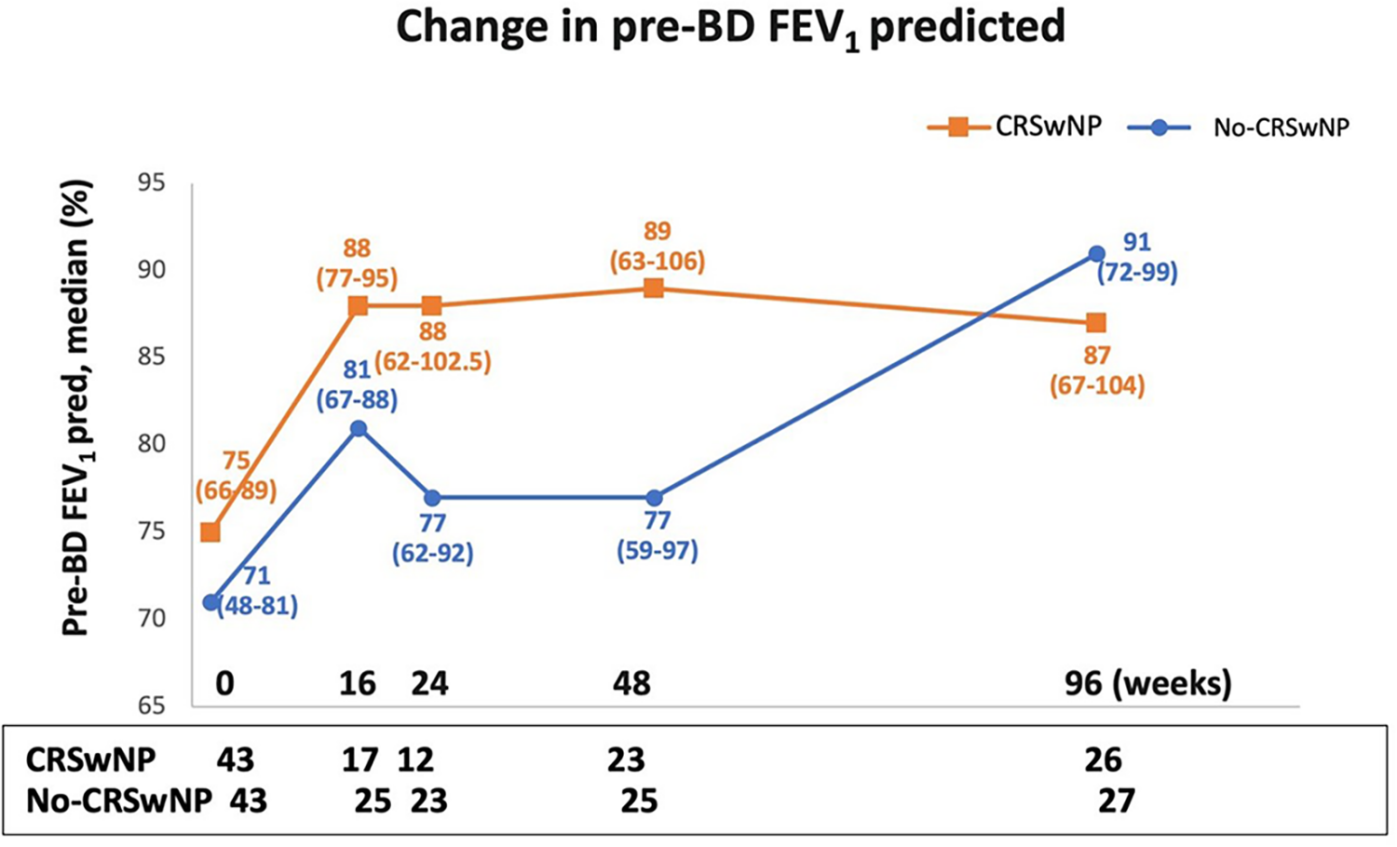
Change in pre-BD FEV_1_ pred. (%) during benralizumab treatment in CRSwNP and No-CRSwNP patients. Median (range) of pre-BD FEV_1_ pred. (%) is reported at the index date and after 16, 24, 48 and 96 weeks of treatment with benralizumab.

Figure 3 illustrates the changes in pre-BD FEV_1_ predicted over the course of benralizumab treatment. Up to 48 weeks, the CRSwNP population showed improvements compared to No-CRSwNP group [89.0% (63.0–106.0) vs. 77.0% (59.0–97.0), respectively]. Remarkably, CRSwNP patients achieved a median pre-BD FEV_1_ predicted value of 88.0% (77.0–95.0) as early as 16 weeks, which then plateaued and remained largely stable until week 96, with values equal or greater than 87.0% throughout the treatment period.

In contrast, the No-CRSwNP group experienced a more gradual improvement, with lower pre-BD FEV_1_ predicted median values than CRSwNP patients [81.0% (67.0–88.0) and 77.0% (62.0–92.0) at 16 and 24 weeks, respectively]. However, by week 96, the No-CRSwNP group reached pre-BD FEV_1_ predicted values similar to the CRSwNP group [91.0% (72.0–99.0)].

### 3.4 OCS-sparing effect

**Figure 4:**
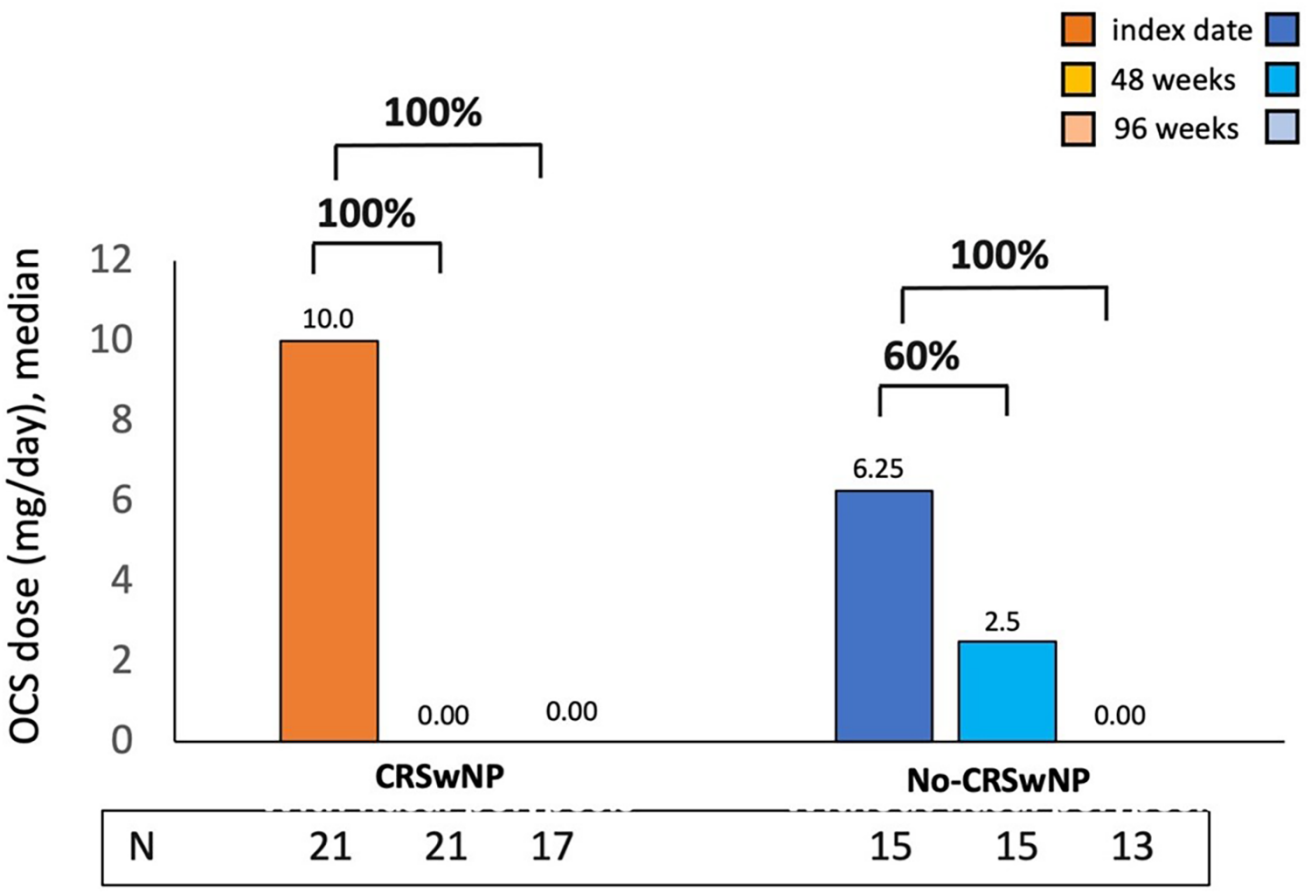
OCS reduction during benralizumab treatment in CRSwNP and No-CRSwNP patients. The median (IQR) OCS daily dose (mg of prednisone equivalent) is reported at the index date and after 4, 16, 24, 48 and 96 weeks of treatment with benralizumab.

Benralizumab OCS-sparing effect was evaluated in CRSwNP and No-CRSwNP groups (Figure 4). At 48 weeks, CRSwNP patients exhibited a median OCS dose of 0.0 mg/day, indicating a 100% reduction from the median dose of 10.0 mg/day used at the index date. During the same timeframe, No-CRSwNP patients reduced their median OCS dose to 2.5 mg/day (60% reduction from the starting dose of 6.25 mg/day). By week 96, both groups achieved a 100% reduction in median OCS dose.

The proportion of patients who reduced and/or eliminated the use of OCS during benralizumab treatment was examined and is shown in Table 2. At 48 weeks, 61.9% of CRSwNP patients and 46.6% of No-CRSwNP patients had completely discontinued OCS treatment; these percentages further increased at 96 weeks, reaching 70.5% and 53.8% in the CRSwNP and No-CRSwNP groups, respectively. Overall, 76.4% of CRSwNP patients and 61.5% of No-CRSwNP patients reduced the OCS daily dose to some extent by 96 weeks. Consistently, a smaller percentage of patients in both groups showed no reduction in OCS use, with slightly higher percentages observed in the No-CRSwNP group (*N* = 7, 46.7% at 48 weeks; *N* = 5, 38.5% at 96 weeks) compared to the CRSwNP group (*N* = 6, 28.6% at 48 weeks, *N* = 4, 23.6% at 96 weeks) (Table 2).

**Table 2:** OCS dose reduction and/or interruption in CRSwNP and No-CRSwNP patients during benralizumab treatment. Data were collected after 48 (A) and 96 weeks (B) of benralizumab treatment. OCS dosage is expressed in daily mg (prednisone equivalent). Data are expressed as *N* (%).

Extent of OCS reduction from index date	48 weeks		96 weeks	
	CRSwNP	No-CRSwNP	CRSwNP	No-CRSwNP
	(*N* = 21)	(*N* = 15)	(*N* = 17)	(*N* = 13)
	*N* (%)	*N* (%)	*N* (%)	*N* (%)
Interruption	13 (61.9)	7 (46.6)	12 (70.5)	7 (53.8)
Any reduction	15 (71.4)	8 (53.3)	13 (76.4)	8 (61.5)
(including interruption)				
≥90% dose reduction	13 (61.9)	7 (46.6)	12 (70.5)	7 (53.8)
≥75% dose reduction	13 (61.9)	7 (46.6)	12 (70.5)	7 (53.8)
≥25% dose reduction	15 (71.4)	7 (46.6)	13 (76.4)	7 (53.8)
No reduction	6 (28.6)	7 (46.7)	4 (23.6)	5 (38.5)

### 3.5 Asthma control

**Figure 5:**
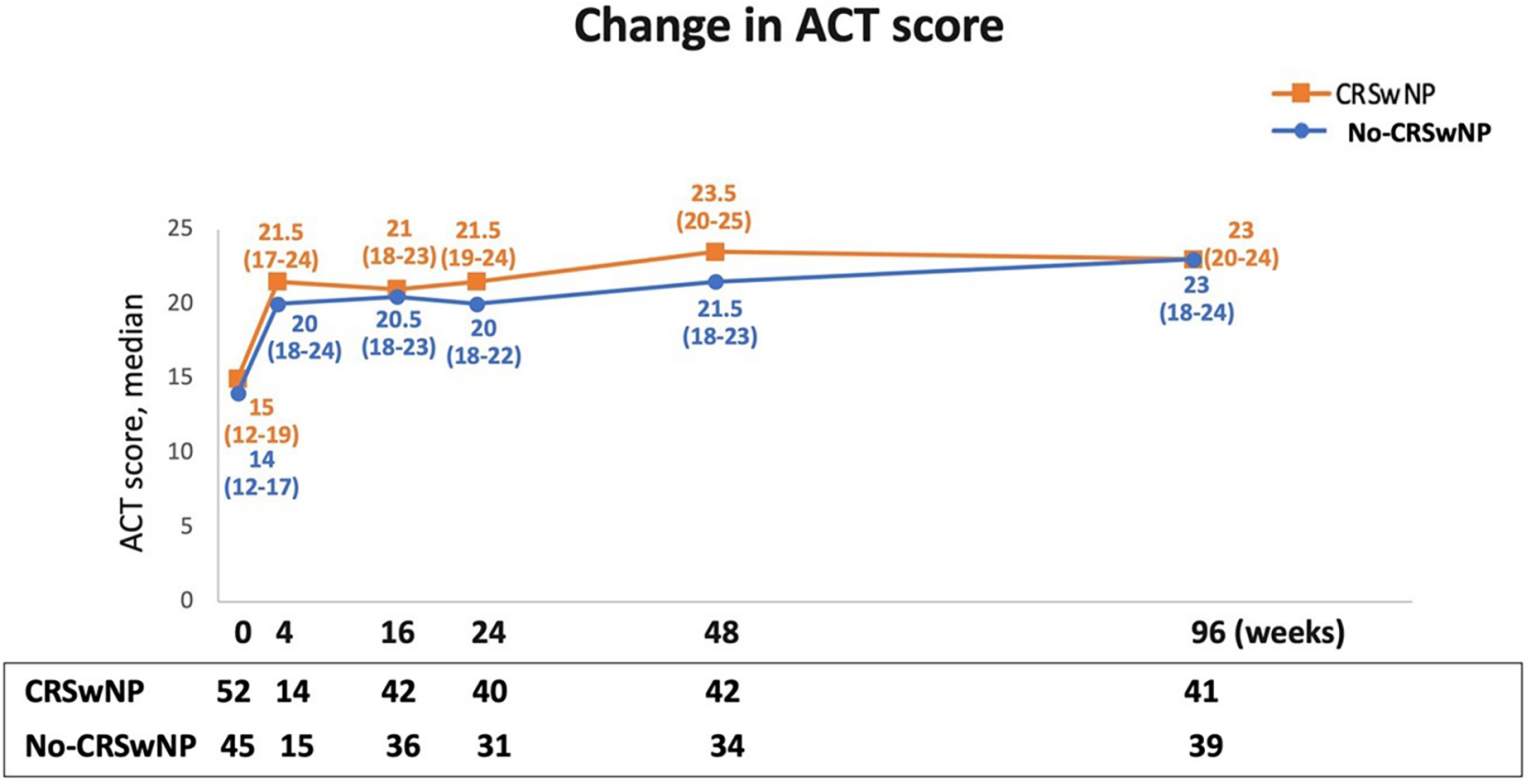
Improvement in asthma control during benralizumab treatment in CRSwNP and No-CRSwNP patients. Change in ACT score is expressed as median (IQR). Data are reported at index date and after 4, 16, 24, 48, and 96 weeks of treatment with benralizumab.

Changes in asthma control were monitored via the ACT during benralizumab treatment. As depicted in Figure 5, the ACT score improved and increased rapidly after just 4 weeks of benralizumab treatment, reaching median values equal to or greater than 20 in both groups [CRSwNP, 21.5 (17.0–24.0)] and No-CRSwNP, 20.0 [18.0–24.0]). Between 16 and 48 weeks, median ACT scores were slightly higher in CRSwNP patients [from 21.0 (18.0–23.0) at 16 weeks to 23.5 (20.0–25.0) at 48 weeks], compared to No-CRSwNP patients [from 20.5 (18.0–23.0) at 16 weeks, to 21.5 (18.0–23.0) at 48 weeks]. However, at 96 weeks, both groups reached identical median ACT scores [CRSwNP, 23.0 (20.0–24.0); No-CRSwNP, 23.0 (18.0–24.0)].

**Figure 6:**
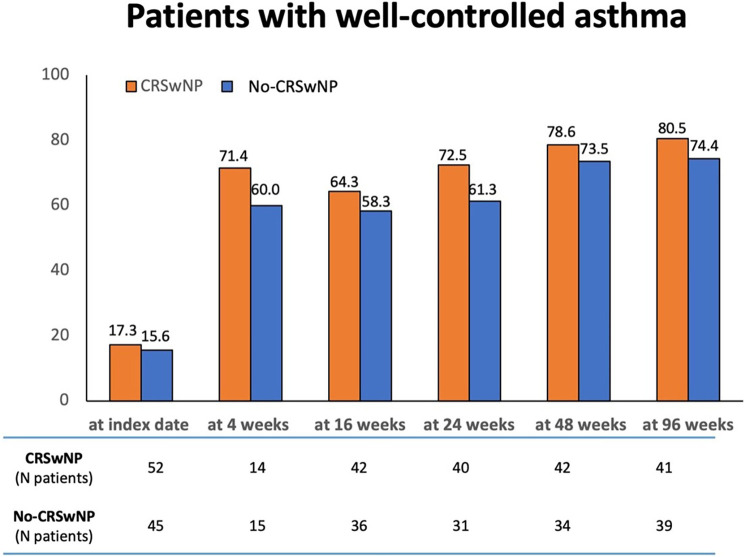
Percentages of patients achieving a well-controlled asthma (ACT score ≥20) in CRSwNP and No-CRSwNP groups at different timepoints. Data are reported at index date and after 4, 16, 24, 48, and 96 weeks of treatment with benralizumab.

Figure 6 shows the proportions of patients achieving a good control of asthma (ACT score ≥20). Compared to patients in the No-CRSwNP group, higher percentages of CRSwNP patients achieved a well-controlled asthma at all considered time points, with up to 80.5% of CRSwNP patients having an ACT score ≥20 registered at 96 weeks. The percentages of No-CRSwNP patients with well-controlled asthma were consistently lower at all time points (up 74.4% at 96 weeks) (Supplementary Table S1).

### 3.6 Discontinuation

At 96 weeks, a similar percentage of CRSwNP and No-CRSwNP patients discontinued benralizumab treatment (10.2% and 11.1%, respectively). Reasons for discontinuation included lack of clinical efficacy (6.8% vs. 3.7%, respectively), adverse events (1.7% vs. 1.9%, respectively), allergic rhinitis (0 vs. 1.9%, respectively), and patient decision (0 vs. 3.7%, respectively). Data are shown in Supplementary Table S2.

## 4 Discussion

This *post hoc* analysis, conducted from the real-life ANANKE study, evaluated the clinical characteristics and outcomes in SEA patients with CRSwNP and those without (No-CRSwNP) treated with benralizumab for up to 96 weeks. This work builds upon the study of D'Amato et al., which assessed the efficacy of benralizumab in reducing symptoms, exacerbations, OCS use and improving lung function and asthma control in the same populations after a median exposure to benralizumab of 9.8 months (27).

Previous research has provided evidence supporting the rapid effectiveness of benralizumab in real-world settings for patients with SEA. While the ANANKE study described asthma outcomes in patients treated for up to 96 weeks (28), only recently benralizumab sustained effectiveness was confirmed over a period of 3 years (29). Recently, the real-world XALOC-1 programme demonstrated that the SEA patients treated with benralizumab had substantial improvements in clinical outcomes irrespective of previous biologic use (30). While some studies have investigated the effectiveness of benralizumab in patients with both SEA and CRSwNP, most of them reported results for treatment periods of up to 24 weeks (22, 23, 31). In the retrospective ORBE II cohort study, Padilla-Galo et al. investigated the effects of benralizumab in SEA patients with and without CRSwNP over 1-year period (32). In addition, Santomasi et al. demonstrated that benralizumab exerted beneficial effects on both SEA and CRSwNP outcomes in comorbid patients treated over a period of 20 months (33). Recently, Pelaia et al. assessed the effectiveness of benralizumab in patients treated up to 2 years. Although the authors did not compare treatment outcomes between CRSwNP and No-CRSwNP patients, their results highlight that CRSwNP patients are more likely to achieve clinical remission (24). Therefore, our study complements previous findings by providing a comprehensive 96-week comparison of benralizumab effectiveness on asthma outcomes in SEA patients with and without CRSwNP for up to 96 weeks.

A close examination of the clinical characteristics of the two patient populations in this study reveals several key findings. Firstly, the age at asthma onset was similar between the two groups. However, CRSwNP patients had a longer duration of asthma and SEA compared to those without CRSwNP, along with a higher BEC. Although the rate of comorbidities (excluding CRSwNP) was comparable between CRSwNP and No-CRSwNP patients, EGPA was more frequent in the CRSwNP group. CRSwNP patients appeared to experience fewer severe exacerbations and exhibited better lung function compared to No CRSwNP patients. A higher proportion of CRSwNP patients used OCS compared to No CRSwNP patients, potentially contributing to the observed differences in exacerbation control and lung function. These findings are in line with existing literature indicating that patients with both SEA and CRSwNP may require a more complex management compared to patients with SEA (13–15). In this study, the sustained effectiveness of benralizumab is evidenced by the decrease in AER observed in both patient cohorts at 96 weeks. When comparing the percentages of AER reduction between the two groups (both any and severe AER), benralizumab appeared to be slightly more effective in CRSwNP patients, at both 48 weeks and 96 weeks, with the greatest difference observed at 48 weeks in severe AER (98.8% in CRSwNP vs. 89.5% in No-CRSwNP). The different response to benralizumab was even more pronounced when considering the percentage of patients free from exacerbations. At 96 weeks, 100% of CRSwNP patients were free from severe exacerbations, compared to 90.7% of No-CRSwNP patients; similarly, 88.1% of CRSwNP patients were free from any exacerbations, compared to 66.7% of No-CRSwNP patients. These findings are consistent with the work by Bagnasco et al., in which exacerbation mean reduction was significantly greater in CRSwNP patients than No-CRSwNP patients (23). In the ORBE II study, patients with and without CRSwNP had a similar reduction in the mean number of severe exacerbations; however, a greater percentage of patients with CRSwNP had at least a 50% reduction in exacerbations (32). Our results are also in line with *post hoc* analyses of the SIROCCO and CALIMA clinical trials, in which the presence of CRSwNP was first identified as a consistent predictor of benralizumab response in terms of exacerbations and other asthma outcomes (20).

The pulmonary function (measured as pre-BD FEV_1_% predicted) improved already at 16 weeks in both populations. At 48 weeks, CRSwNP patients improved pre-BD FEV_1_ predicted values compared with No-CRSwNP patients; only at 96 weeks, the two populations reached similar values. These findings suggest that benralizumab may exert rapid effects in patients with CRSwNP, with improvement in pulmonary function observed as early as 16 weeks and maintained throughout the observation period. Conversely, the response to benralizumab in patients without CRSwNP appeared to be more gradual. A plateau was not reached, but a peak was observed at 96 weeks. These findings are consistent with other real-life observations, which showed that benralizumab has a more rapid (22) and pronounced effect on respiratory parameters in CRSwNP than No-CRSwNP patients (22, 32). In contrast, Bagnasco et al. reported a greater improvement in both volume and predicted volume of pre-BD FEV_1_ in patients without CRSwNP than patients with CRSwNP after 24 weeks of treatment (23). Differences in the length of observation period and clinical characteristics at baseline may account for these discrepant results (23).

A profound OCS-sparing effect of benralizumab was observed in all patients of our study. Although both groups were characterized by a median OCS reduction of 100% at 96 weeks, the action appears to be more pronounced in CRSwNP patients, as this group used a higher median OCS dose at the index date (10 mg) compared with No-CRSwNP patients (6.5 mg) (22, 23, 32).

Both CRSwNP and No-CRSwNP populations achieved asthma control already at 4 weeks after starting benralizumab. This positive outcome persisted throughout the study, with both groups maintaining well-controlled asthma for up to 96 weeks, as evidenced by median ACT scores ≥20. While median ACT scores were almost identical between the two populations, CRSwNP group featured the highest percentage of patients with ACT ≥ 20 at all time points considered up to 96 weeks, compared to No-CRSwNP group. This observation suggests that the presence of CRSwNP may contribute to a more favorable response to benralizumab in terms of asthma control. This finding gains even greater relevance in light of the well-established negative interplay between CRSwNP and asthma; regardless of the presence of nasal polyps, benralizumab could effectively control asthma symptoms, leading to a meaningful improvement of ACT score. Importantly, our results are in line with other real-life studies. Among these, Padilla-Galo et al. showed that a greater proportion of CRSwNP patients (76.9%) achieved a clinically meaningful improvement in asthma control (defined as an increase in ACT score of ≥3 points), compared to patients without CRSwNP (70.0%) (32).

This study describes the long-term improvement of asthma outcomes in CRSwNP and No-CRSwNP patients treated with benralizumab. At 96 weeks, CRSwNP and No-CRSwNP responded similarly to benralizumab; however, more pronounced effects and rapid onset of action have been noticed for all outcomes in CRSwNP compared to No-CRSwNP patients. Differences in clinical characteristics at baseline (i.e., more frequent exacerbations and worse respiratory function in No CRSwNP vs. CRSwNP patients) may have contributed to the enhanced response to benralizumab observed in the CRSwNP population. Nevertheless, previous studies have reported similar results. Since the No CRSwNP group consisted predominantly of female patients, whereas the CRSwNP group had a balanced male-to-female ratio, we cannot exclude the potential influence of this gender imbalance between the two groups on the observed differences in benralizumab effectiveness. Indeed, female sex hormones may play a role in asthma pathogenesis and have been associated with poor outcomes (34). To date, few studies have investigated the impact of gender on biologics treatment response, and existing evidence suggests that gender is not a determining factor in treatment effectiveness (35).

In addition to the literature discussed above, the 24-week data obtained by Nolasco et al. potentially suggest that patients with CRSwNP may experience a quicker response to benralizumab compared to those without (22). Recently, benralizumab capacity to attenuate eosinophilic inflammation has been associated with its ability to restore NK cell function independently of its OCS-sparing effect. In addition, the recovery of NK cell activity exhibited a positive correlation with improvements in FEV_1_. Restoring NK cell function not only helps improve respiratory function, but also strengthens the body's defenses against infections and relapses, which can trigger severe asthma attacks. This dual benefit highlights how benralizumab can effectively manage severe eosinophilic asthma (18). This unique mode of action of benralizumab may provide a more comprehensive approach to restoring immune balance and achieving asthma control in SEA patients with CRSwNP even in the most difficult to manage patients, such as those with CRSwNP. This potential for enhanced effectiveness is supported by the observation that CRSwNP patients exhibit more severe asthma characteristics at the index date, as evidenced by their higher OCS use, compared to the No-CRSwNP group. The excessive eosinophilic accumulation and T2 inflammation characterizing comorbid patients could further explain the superior and faster effects observed with benralizumab treatment (36). Therefore, the presence of CRSwNP may amplify the positive effects of benralizumab's broad mechanism of action.

This study has a few limitations, the main ones represented by its retrospective observational design, the lack of a control arm and its restriction to data available in routine clinical practice. Nevertheless, this study provides the first long-term, real-world insights into the utilization and effectiveness of benralizumab in a large cohort of SEA patients with and without CRSwNP in Italy. Secondly, the retrospective design and the extended follow-up period have contributed to the loss of patients observed during the follow-up. As highlighted in previous publications (26–28), the absence of statistical analysis represents a considerable drawback in all the ANANKE studies, although it does not diminish the relevance of the observations. Indeed, ANANKE is an observational retrospective study whose primary endpoint is to describe the clinical profile of patients eligible for treatment with benralizumab in a real-world setting; therefore, the analyses of the variables were descriptive only, and no formal hypotheses were pre-specified and tested.

## 5 Conclusions

This study explores for the first time the long-term impact of benralizumab on patients with SEA and CRSwNP, compared to those without CRSwNP. The results not only reinforce the established effectiveness of benralizumab in SEA with CRSwNP (25), but also suggest a potentially rapid onset of action in this specific patient population. Further research is warranted to confirm this observation and to understand the mechanisms underlying benralizumab superior efficacy and rapid action in the presence of CRSwNP.

Overall, these results emphasize the importance of considering the presence of comorbidities when making therapeutic decisions, further highlighting the importance of tailored treatment approaches based on individual patient characteristics, ultimately optimizing clinical outcomes in the management of severe asthma.

## Data Availability

The data analyzed in this study is subject to the following licenses/restrictions: The datasets used and/or analyzed during the current study are available from the corresponding author upon reasonable request. Requests to access these datasets should be directed to marielladam@hotmail.it.
