# Supplementary material for: Durability of benralizumab effectiveness in severe eosinophilic asthma patients with and without chronic rhinosinusitis with nasal polyps: a post hoc analysis from the ANANKE study
Source: Front Allergy. 2025 Mar 20;6:1501196. doi: 10.3389/falgy.2025.1501196 (PMC11965627; doi:10.3389/falgy.2025.1501196)
Supplement: Supplementary file 1 [file Table1.docx]

**Additional files**

|  | **CRSwNP** | **No-CRSwNP** |
| --- | --- | --- |
| **at index date (N=52,45)** | 9 (17.3%) | 7 (15.5%) |
| **at 4 weeks (N=14,15)** | 10 (71.4%) | 9 (60.0%) |
| **at 16 weeks (N=42,36)** | 27 (64.2%) | 21 (58.3%) |
| **at 24 weeks (N=40,31)** | 29 (72.5%) | 19 (61.2%) |
| **at 48 weeks (N=42,34)** | 33 (78.5%) | 25 (73.5%) |
| **at 96 weeks (N=41,39)** | 33 (80.5%) | 29 (74.4%) |

**Supplementary Table 1. Patients achieving well-controlled asthma (i.e. ACT score >=20) during benralizumab treatment.** Data are expressed as N (%).

|  | **CRSwNP (N=59)** | **No-CRSwNP (N=54)** |
| --- | --- | --- |
| **Patients experiencing discontinuation prior to 96 weeks** | 6 (10.2) | 6 (11.1) |
| **Reasons for benralizumab permanent discontinuation within 96 weeks** |  |  |
| **Lack of clinical efficacy** | 4 (6.8) | 2 (3.7) |
| **Adverse events** | 1 (1.7) | 1 (1.9) |
| **Other (ALLERGIC RHINITIS)** | 0 | 1 (1.9) |
| **Patient decision** | 0 | 2 (3.7) |

**Supplementary Table 2. Proportions of CRSwNP and No-CRSwNP patients experiencing discontinuation prior to 96 weeks of benralizumab treatment.** Data are expressed as N (%).
